# Personal

**Published:** 1908-09

**Authors:** 


### PERSONAL.

Dr. Jane Wall Carroll, of Buffalo, was elected president of the medical section of the National Fraternal Congress, recently convened at Put in Bay, Ohio.

Dr. Howard J. Rogers, of Albany, first assistant commissioner of education of the State of New York, according to an Associated Press despatch of August 22, 1908, has resigned his office to devote his time to mining interests in the west. Dr. Rogers was in charge of universities, colleges, professional and technical schools, and the execution of the laws concerning the profession, also the relations and chartering of higher institutions. His resignation will be greatly regretted by all who had occasion to meet him officially, as well as by a large circle of personal friends.

Dr. Carl G. Leo-Wolf, of Niagara Falls, has returned from a two months' trip abroad, and has resumed his professional practice.

Dr. L. Bradley Dorr, of Buffalo, has been nominated for Congress by the 35th congressional district republican convention. Dr. Dorr is one of the prominent practitioners in this city and would represent the district with credit should the nomination be ratified at the polls.

Dr. Carl S. Tompkins, of Randolph, N. Y., has taken apartments in the Jupp apartment house, corner Allen street, and Elmwood avenue, Buffalo, which he and Mrs. Tompkins will occupy after the first of September.

Dr. James L. Gallagher, one of the staff physicians of the Emergency Hospital, Buffalo, has been elected supreme medical examiner of the Ancient Order of Foresters of the High Court of the United States.

